# Understanding the context of delays in seeking appropriate care for children with symptoms of severe malaria in Uganda

**DOI:** 10.1371/journal.pone.0217262

**Published:** 2019-06-05

**Authors:** Arthur Mpimbaza, Susan Nayiga, Grace Ndeezi, Philip J. Rosenthal, Charles Karamagi, Anne Katahoire

**Affiliations:** 1 Child Health & Development Centre, Makerere University, College of Health Sciences, Kampala, Uganda; 2 Infectious Diseases Research Collaboration, Kampala, Uganda; 3 Department of Pediatrics & Child Health, Makerere University, College of Health Sciences, Kampala, Uganda; 4 Department of Medicine, University of California, San Francisco, CA, United States of America; 5 Clinical Epidemiology Unit, Department of Medicine, Makerere University, College of Health Sciences, Kampala, Uganda; Centers for Disease Control and Prevention, UNITED STATES

## Abstract

**Introduction:**

A large proportion of children with uncomplicated malaria receive appropriate treatment late, contributing to progression of illness to severe disease. We explored contexts of caregiver delays in seeking appropriate care for children with severe malaria.

**Methods:**

This qualitative study was conducted at the Children’s Ward of Jinja Hospital, where children with severe malaria were hospitalized. A total of 22in-depth interviews were conducted with caregivers of children hospitalized with severe malaria. Issues explored were formulated based on the Partners for Applied Social Sciences (PASS) model, focusing on facilitators and barriersto caregivers’promptseeking and accessing ofappropriate care. The data were coded deductively using ATLAS.ti (version 7.5). Codes were then grouped into families based on emerging themes.

**Results:**

Caregivers’ rating of initial symptoms as mild illness lead to delays in response. Use of home initiated interventions with presumably ineffective herbs or medicines was common, leading to further delay. When care was sought outside the home, drug shops were preferred over public health facilities for reasons of convenience. Drug shops often provided sub-optimal care, and thus contributed to delays in access to appropriate care. Public facilities were often a last resort when illness was perceived to be progressing to severe disease. Further delays occurred at health facilities due to inadequate referral systems.

**Conclusion:**

Communities living in endemic areas need to be sensitized about the significance of fever, even if mild, as an indicator of malaria. Additionally, amidst ongoing efforts at bringing antimalarial treatment services closer to communities, the value of drug shops as providers ofrationalantimalarialtreatment needs to be reviewed.

## Introduction

For African children with uncomplicated malaria, the World Health Organization (WHO) recommends prompt treatment with artemisinin-based combination therapies (ACTs) within 24 hours of illness onset as a key strategy for preventing progression to severe malaria [1]. Unlike in the past, when ACTs were scarce and expensive [2], efforts by governments, international organizations, and partner institutions have resulted in increased availability of high quality and affordable ACTs in both the private and public health sectors [3]. This achievement has likely contributed greatly to reductions in malaria morbidity and mortality in Africa [4, 5]. However, despite increases in ACT coverage rates, the proportion of eligible children receiving ACTs within 24 hours of illness onset remains low [6], partly explaining why hundreds of thousands of children continue to die of malaria each year [7].

If malaria control and elimination programs are to succeed, there is a need to ensure maximum treatment effectiveness, characterized by high quality case management and appropriate health seeking behavior by patients or caregivers [8]. Studies in sub-Saharan Africa have shown that the delivery of antimalarials may fail at different stages, including at the initiation of treatment at home, or due to health care providers offering ineffective medicines [9]. For children, caregivers’ response to illness is the first and probably the most important step in determining if treatment is received promptly [10]. Circumstances surrounding caregivers’ delays in seeking appropriate care are complex [11]. Knowledge, perception, and beliefs of illness are known to influence the time taken to appraise symptoms, thus influencing response time and potentially leading to delays in seeking care.

Categorization of fever as a minor ailment by caregivers has been shown to delay response to illness among children with malaria in Tanzania [12]. Treatment of illness episodes with local remedies and herbs contributes to further delays in seeking appropriate care [13, 14]. Furthermore, if care is sought at drug shops, where the quality of care is often sub-optimal [15], delay in receiving appropriate treatment is a likely outcome [16, 17]. Social networks, including caregiver partners, parents, in-laws, and neighbors involved in negotiating response to illness can also be associated with delays in care [18].

Understanding caregivers’ health seeking behavior while taking into consideration contextual factors that govern behavior is a prerequisite for formulating policies targeted at optimizing response to illness [19]. However, policy efforts continue to focus on provider factors such as increasing availability of ACTs to communities at risk, while neglecting demand side factors such as how caregivers interpret and respond to symptoms of illness. Sensitizing communities to availability of services and providing classical biomedical descriptions of symptoms of malaria have had little impact on caregivers seeking care promptly [20]. This situation is likely explained by consideration of care seeking as a linear pathway, neglecting social and environmental contexts that govern individual behavior and response types which shape pathways to care[20–22]. Utilizing interviews with caregivers of children who presented with severe malaria in Jinja, Uganda, we explored the social and environmental constraints that shape when and how caregivers respond to illness, and sought to explain delays in accessing appropriate treatment for malaria.

### Research team

A social scientist with undergraduate and postgraduate training in social science, and epidemiology, respectively led all interviews. The lead interviewer is female and has experience spanning a period of 10 years, working as a coordinator on several qualitative studies [23–25]. A member of the local hospital staff, who was well accustomed with local language, culture, and social norms of the locals recruited patients, explaining to respondents reasons for conducting the study, introducing the interviewer and when applicable translating and responses. At the time of conducting interviews, the interview team was aware of the main findings of the primary study. Prior to conducting the interview, the interview team introduced themselves to the study participants highlighting, who they were, and where they came from. Additionally, they explained the purpose of the interview to study participants.

## Methods

### Study design

This was a qualitative study, linked to a study of determinants of severe malaria in children, in which delay in seeking appropriate care was found to be a strong risk factor for severe malaria [16]. Caregivers of children hospitalized with severe malaria were interviewed to explore how their social and environmental contexts influenced when and how they responded to illness. We explored motivations for caregivers’ actions in response to illness, explaining delays in seeking and accessing appropriate treatment for their children. Our approach was mainly deductive in nature, driven by the hypothesis that delay in seeking appropriate care was a risk factor for severe malaria.

### Theoretical framework

To explore contextual factors, this study drew on the Partners for Applied Social Sciences (PASS) model [26]. The model is built on the backbone ofa pathway model, focusing on factors involved ineach step of the pathway to care that hinder or facilitate prompt seeking or access to appropriate care. Factors in the model are grouped into four main categories: 1) illnessperception and explanatory models; 2) social values; 3) access tocare and resource seeking; and 4) medicalpluralism [26]. Considering social and environmental contexts, we explored how these factors contributed to caregiver delays in seeking appropriate care for children with malaria.

### Study setting

The study was conducted in the catchment area of the Children’s Ward of Jinja Regional Referral Hospital, which includes 10 districts located in the Busoga sub-region, with a few patients also coming from two districts located in the Buganda region [16]. Malaria transmission in the sub-region is considered hypo to hyper endemic [27]. In 2016, the malaria parasite prevalence rate among children in the Busoga sub-region was 53.1% based on an HRP2-basedrapid diagnostic test [28]. The economic base for 53% of the local people is subsistence-oriented farming [29], with some villagers engaging in small-scale business such as operating small roadside shops. The Busoga region has 42.0% of the population living in poverty and is known to suffer from famine [29], malnutrition, very high infant mortality rates, and rampant jigger epidemics [30, 31]. Literacy levels are low, with 56.1% and 43.8% of male and female heads of households being literate. The Early Childhood Development Index, a composite measure of the developmental status of children, was reported to be 55.5% in the region, the lowest value reported in Uganda [28].

The local health arena in Busoga is pluralistic, with access to traditional medicine and biomedicines at both public and private facilities [32]. The region has public health facilities ranging from level II to level IV health centers, district hospitals and a regional referral hospital. The average distance between level III (and above) health facilities and households is < 5 km [16]. Services rendered at public facilities are plagued by staff absenteeism and frequent stock outs of essential medicines [33]. Drug shops, though popular, often offer substandard services [15, 34, 35]. Initiatives at improving quality of services at drug shops have been implemented in the region, with reported success [36].

### Sampling

Sampling was purposive and research participants were selected based on gender, relation to the child, age, time taken to seek care, and form of severe malaria. Maximum variation in the selection of respondents ensured that different social groups were represented, including consideration of employment and socio-economic status. The study team approached eligible respondents during hospitalization at Jinja Children’s Hospital. Respondents were purposively included in the sampling frame.

### Sample size

The final sample size for the study was 22 caregivers and was not pre-determined, but reached based on the principle of data saturation, where gathering fresh data from additional respondents did not reveal new insights or findings based on our theoretical model. All eligible study participants agreed to participate when approached.

### Setting of data collection

Interviews with caregivers were conducted privately during the recovery period of the child’s hospitalization either in the hospital gardens or in one of the study offices, depending on the respondent’s choice. Both venues offered privacy, with minimum interruption. For all 22-interview sessions, no one other than the child’s primary caregiver and interviewers were present.

### Data collection

An interview guide was developed (**S1 Appendix**) based on key themes agreed upon by the study team based on observations noted during the primary study. The interview guide was pilot tested and modified by the lead interviewer, improving on sequencing of questions. Interviews lasted less than 2 hours and were conducted in the local language. The interviews were audio recorded and transcribed in the language in which they were conducted before translation into English by an experienced interviewer. The interviewer did not repeat any of the interviews, nor were transcribed interviews returned to study participants for comment and correction. Additionally, upon completion of each interview, the interviewer noted a summary of key observations during the interview. Patient responses were categorized into three phases: 1) illness recognition, 2) response to illness and 3) actions.

### Definition of terms

Delay was defined as a prolonged interval (typically >24 hours) between illness onset and commencement of appropriate treatment. Illness recognition was defined as the caregivers’ appraisal of symptoms as a sign of illness. Response was defined as the caregiver acknowledging the need to intervene upon the child’s illness. Caregivers’ actions were defined as the measures taken in response to illness. Individuals who provided advice to the caregiver in terms of how to respond to the child’s illness were referred to as the therapeutic management group[26]. The primary caregiver was the person who was with the child right from onset to presentation to hospital.

### Data analysis

Analysis of data was deductive in nature, informed by preliminary findings of the primary study that identified delayed seeking of care and seeking care at drug shops as potent risk factors for severe malaria [16]. Upon completion of the primary study, findings were presented to a panel of experts, including pediatricians, social scientists, and medical anthropologists, during which themes worthy exploration were formulated. Individual transcripts were entered into ATLAS.ti. Version 7.5.18 (Atlas.ti. GmbH, Berlin Parts), and coded (a code was a word or short phrase that symbolically assigned meaning to a portion of language in the data) by the lead investigator of the study under the supervision of a medical anthropologist using a mixed approach of both open and closed coding based on pre–defined criteria. Related codes were categorized into families’ representative of different themes informed by the findings of the quantitative study [16]. These themes formed the basis of the write-up and discussion. Participants were not involved nor consulted for opinion on themes.

The design and findings of this study are reported following the consolidated criteria for reporting qualitative research (COREQ; **S2 Appendix**;**) [37]**

### Ethics approval

Institutional approval was obtained from the Uganda National Council of Science and Technology, and the Institutional Review Boards from the College of Health Sciences, Makerere University, and the University of California San Francisco. Written informed consent was obtained from each caregiver. In addition to obtaining consent, the interviews were conducted in accordance with good clinical practice, applicable patient privacy requirements, and the guiding principles of the Declaration of Helsinki.

## Results

### Characteristics of the study sample

We interviewed 22 caregivers, each with a child hospitalized with severe malaria at the Children’s Ward of Jinja Regional Referral Hospital during the months of February and March 2016.The majority of the caregivers were mothers (15, 68.2%), with grandmothers (3, 13.6%), fathers (2, 9.1%), aunts (2, 4.5%), and stepmothers (1, 4.5%) accounting for the others. The average age of caregivers’ was 31 years (range 18 to 48years). Most (68%) caregivers were primarily engaged in subsistence farming, with occasional selling of excess food (Table 1).

**Table 1:** Demographic and clinical characteristics of caregivers and their enrolled children.

**Characteristics of caregivers of enrolled children**		
Average age in years		31.6
Age range in years		18 to 48
Relation with child	Mother	114 (63.6%)
	Grandmother	1 (4.5%)
	Stepmother	2 (9.0%)
	Father	2 (9.0%)
	Grandmother	3 (13.6%)
Occupation	Peasant	15 (68.1%)
	Business	5 (22.7%)
	Tailor	1 (4.5%)
	Waitress	1 (4.5%)
**Characteristics of enrolled children**		
Age in years		2.6
Age range in years		0.5 to 7.5
Gender Female		9 (40.9%)
Type of severe malaria	Unconsciousness	5 (22.7%)
	Severe anemia	10 (45.4%)
	Respiratory distress	3 (13.6%)
	Unconsciousness & Severe anemia	1 (4.5%)
	Respiratory distress & Severe anemia	1 (4.5%)
	Respiratory distress & Unconsciousness	1 (4.5%)
	Respiratory distress & Unconsciousness& Severe anemia	1 (4.5%)

### Different kinds of fevers

Caregivers reported that their children experienced different kinds of fevers, characterized as mild or severe, sometimes contributing to delays in seeking care outside of the home as some of the fevers were ranked as low grade, not deserving serious attention (Quotes A317, A324).

***A317:** “If a child has a temperature… you get a damp cloth and sponge them…*.*for the simple one you give panadol, they sweat and the fever goes down”*

***A324:**“The body may be hot but not very hot. The child may be playing and eating. We can say that let us give them some coartem. They will get better*.*”*

Fever described as “hot "or “high “in isolation or with other symptoms such as loss of appetite or extreme body weakness was considered a sign of serious illness, triggering rapid response (Quotes A316, A308).

***A316:**“The very high fever can cause me to run. If I delay, the child weakens. If I delay, the child gets dehydrated. If I delay it makes him anemic*.*"*

***A308:** “When he is all hot? No, at least you put a cloth in water, squeeze the water out and wipe him from head to toe…then proceed. Because at that point it is excessive to the extent that both the hands and the legs are hot. It is excessive and you have to head straight to the hospital*.*”*

### Caregivers understanding of malaria

Caregivers also talked about mild and severe malaria, with some equating the occurrence of fever with malaria. They mentioned symptoms of severe malaria such as anemia that they considered could result in death and indeed, when children had these symptoms caregivers responded promptly.

*A311: “…..he can get malaria, gets weak, blood gets low, water in his body gets drained. So if you stay home and ignore the situation and say I was given this let me just wait the child can die in that way*.*”*

Furthermore, caregivers explained that severe malaria was a result of delayed seeking of appropriate care (Quotes A319, A325, A326).

***A319:**“…. The only problem is that if you delay, the illness gets severe*.*”*

***A326:**“…. If (malaria) gets the child and you delay to go to hospital it has to kill the child because you have delayed*.*”*

These delays however were not necessarily deliberate or within the control of the caregivers.

### Therapeutic management groups as a source of delay

Caregivers explained that the interpretation of the child’s illness sometimes involved other members of the family. Caregivers living with parents or mothers-in-law for example explained that they felt obliged to seek advice from them. Others consulted included fathers, grandparents, neighbors, and friends. The process of consultation itself caused delays. In some cases the symptoms were perceived to be because of witchcraft or other ailments and so herbal medicines were prescribed in place of conventional medicines, thus contributing to delays (Quote A309).

***A309:**“They stopped me from giving her the drug…….the old people including my mother in law*.*”*

Some mothers expressed independence to make decisions without consultation or approval of the fathers (Quote A315).

**A315:** “Recently it happened. He told me to go to Nalufenya and I refused and decided to go to another place because the people at *** hospital delay patients. My child will die from there. They toss you up and down before offering any treatment. In that way we did not agree. I went on my own and he did not give me money. When I got there I called him on phone and informed him of where I was and he came.*”*

### Delays due to home treatment

At home, caregivers often initiated treatment with a combination of herbs and conventional medicines. This was often followed by a waiting period in anticipation of recovery following use of these medicines. This contributed to delays in seeking appropriate care. Conventional medicines provided included panadol, oral antibiotics, and antimalarials, often purchased from drug shops, or left over from treatment of a previous illness episode (Quotes A307).

***A307:** "On Saturday I had some coartem so I picked one coartem and panadol and I gave him in the morning. Then in the evening, I gave him more. I then added him more on Sunday morning and also on Sunday evening. When it came to midnight in the night, he started convulsing. I tried to wipe him a bit. I feared to give him more medicine. I picked a car and brought him to hospital and they treated him from here*.*”*

Use of herbal medicines was common, with 'mululuza,' bombo, ‘and 'lubirizi' being the most frequently mentioned. These were given concurrently with conventional medicines. Herbs were primarily given to reduce fever, rarely with the intention to cure illness (Quote A328).

*A328: “When I am at home, I use a rag to wipe the body so that the temperature goes down. I also get lubirizi and bombo and give the child ….. Now, I first crush them in a cup and give the child to drink then I pour the rest in the child’s basin for the child to bathe………….They relieve the child*.*”*

Mashed onions and garlic smeared on the child's body were used to prevent and cure convulsions; however this intervention did not prevent caregivers from seeking care at public health facilities.

***A312:**“I think that when a child convulses it is malaria. We were told that when he convulses we smear him with onions and it stops*.*”*

### Delays due to drug shops

Caregivers also reported going to drug shops and clinics because of their close proximity, low cost, and availability of medicines.

***A309:**"The government hospital is very far from our home so we first go to the drug shop and then when the condition does not change then we go to the government hospital*.*”*

***A325:**"We take them to the nearby clinic where we can get some drugs. They tell us that when you go there sometimes they find him some drugs. They give us some drugs and I also explain the problems the child has, for this number of days and the illness is like this and they try to mix for me some drugs*.*”*

However, despite being convenient, drug shops often provide sub-optimal care. The inadequacies of drug shops reported by caregivers included the staff’s lack of knowledge and equipment to diagnose malaria. There were delays described in the referral process even when the child was not getting better (Quotes A312, A308).

**A308**:”the private clinic where we started, they ‘ate’* a lot of money. Because whatever they told us, we would do. They would tell you buy this, you would go and buy. They would tell you buy a syrup, you would go and buy. Even others I just left them behind. Because initially they gave us syrup but when I went with them to Lulyambuzi, they sorted them, we went with 7 syrups. New ones. They sorted them and we threw away 5 of them. They left us with 2 and said those are the ones we should use for this illness. Because all the person was chasing back there was money. Just wanting to get money. But we didn’t know what was wrong with the child. But when we gave him those two syrups and threw the 5, he started getting back in line. But when we combined with the others, it would be as if you are increasing the sickness. But he has ‘eaten’ money; I don’t even know the amount. For a long time, this child has not been well.”* A metaphor used to suggest fraud, which in this context implied that providers were extorting money from caregivers for unwarranted services.

***A312:**"I feel like when you go to the drug shop*, *the treatment is expensive.” … If you do not rush them to hospital and they check what the problem is, you may go to the drug shop and they give him treatment that does not relate with malaria and as a result they may die”*

Caregivers explained that they sought care at drug shops for illnesses that they considered mild. Only when symptoms persisted or the child deteriorated, did they opt to seek care at a public health facility, which they deemed more appropriate for treating severe malaria (Quotes A312, A324).

*A312: “It seems what causes them is the same thing but I think the severe one will have grown and would no longer be managed by drug shops and the like but it requires a blood sample to be taken and they see the actual disease and the drugs they will use.*.*”*

*A324: "Where I stay there is a clinic nearby. That is where I usually get first aid. When it gets worse then we come here… …We only get first aid from there. When it gets worse and they cannot manage at the clinic then we go there*.*”*

### Delays at public health facilities

Caregivers explained that public health facilities often lacked medicines, and had rude health workers who were often absent from work (Quote A315).Caregivers expressed frustration regarding spending money on transport to public health facilities that did not have medicines. They further explained that often they were asked to buy medicines at public health facilities, which was a problem for caregivers that did not have money.

***A315:** There is another place but they never have medicine*. *When you go there they tell you that there are no drugs*

***Interviewer*:** *What place is that?*

**A315:** It is called *** health center.

***Interviewer:** It is a government hospital*.

***A315*:** *Yes*

***Interviewer:** Health center three or two*.

***A315:** Three but when you get there they tell you that they do not have drugs. You may get there and there are no health workers*.

***A317**: “Yes, you need transport of 1500 plus it is painful…………..You find that there are no drugs, even panadol they don’t give you that is why we don’t go. So you would use that money and go to the clinic*.*”*

While public health facilities were reported to promptly refer patients, caregivers had to arrange for transport to the next level. This contributed to delays in accessing appropriate care (**S3 Appendix**). Overall, multiple processes contributed to delays in access to appropriate care for children that were eventually diagnosed with severe malaria.

## Discussion

We studied contexts that contributed to delays in appropriate care for children who eventually developed severe malaria. Consistent with previous reports[20], mild fevers led to delays in response. Caregivers responded to what they perceived as mild illness with ineffective home interventions. When care was sought outside the home, drug shops were preferred over public facilities, but because the care was often sub-optimal, there was delay in accessing appropriate treatment.

Caregivers ably explained the cause of malaria and were aware of the common manifestations of severe malaria; appreciating the fact, that malaria could cause death. However, their understanding did not appear to have a bearing on how they responded to illness and on the time to seek appropriate care, suggesting that response were influenced by other factors, such as the contexts within which they lived [20]. Caregivers’ perceptions of illness as mild based on low fever has been reported in other countries in sub-Saharan Africa [38], possibly explained by the subtle, non-specific and irregular nature of initial symptoms of most malaria episodes [39, 40]. In such circumstances, caregivers may feel that a child’s illness is benign, thus opting to observe or provide simple, ineffective home remedies [12, 41], contributing to delay in seeking appropriate care for malaria. Home remedies may suffice for common childhood viral infections, which are often self-limiting [42], but slow response to malaria is dangerous, as established infection can rapidly progress to severe disease [43].

Consultations with family members and neighbors were also a common cause of delay. Advice given was often using of herbs, contributing to delays in seeking appropriate care [18]. Culturally, grandmothers and mothers-in-law, particularly if paternal, hold a dominant position, to the extent that caregivers are obliged to follow advice, even if they disagree [18, 41]. Fathers were consulted, often times for financial support. However, in contrast to previous reports [44], involvement of fathers did not notably contribute to delays in care. Some caregivers reported having autonomy to make decisions in the absence of fathers or when dealing with an uncooperative father, demonstrating empowerment of some mothers [45].

Outside homes, drug shops were the preferred choice of care, a finding consistent with results of other studies in the region [46, 47]. Drug shops have proliferated in both rural and urban areas [3, 47]. As reported previously, short distance from home, lower cost of obtaining care compared to health centers, short waiting times, and ease of accessing medicines were reasons for preference of drug shops [15, 48]. Interestingly, caregivers sought care at drug shops for symptoms deemed mild and not serious, suggesting that they were aware of the shortcomings of these shops. The popularity of drug shops offers an opportunity for promoting timely access to appropriate antimalarial treatment [46, 49]. However, limitations of drug shops, including lack of provider knowledge on management of malaria [36, 50, 51], poor referral practices [52], lack of diagnostic capacity [53, 54], and profit driven behaviors [55], remain major hurdles. Programs directed at improving services at drug shops have yielded mixed results [56]. Publications from the ACT Watch group, a multi-country research project designed to fill evidence gaps in malaria treatment [57], suggest that despite efforts to improve care, appropriateness of antimalarial treatment at drug shops remains sub-optimal [49, 58–66]. In Tanzania, the Accredited Drug Dispensing Outlet program for regulating and training drug shops to provide affordable and quality medicines was successful in promoting appropriate antimalarial treatment [67, 68]. However, sustaining gains has proven to be a problem [69], with reports indicating providers returning to poor dispensing practices [70, 71]. Rapid Diagnostic Tests have been introduced at drugs shops to rationalize use of antimalarials, but amidst resistance from providers given the desire to profit through the sale of anti-malarial treatments, and from caregivers due to high costs [72, 73].

What is the best approach for improving delivery of health services amidst constraints of the public health system and the popular private sector? One approach is to strengthen the public health system which, despite its challenges [74], has the infrastructure and human resources for effective delivery of health services [75]. Beyond focusing on patient care, efforts should to be directed at improving hospitality services at public facilities. Studies in Uganda and Malawi have shown that abusive and disrespectful health workers were barriers to accessing care at public facilities [76, 77]. This may partly explain why caregivers initially seek care at drug shops, whose hospitability services are likely far better due to the need to maintain customers [78, 79]. Strengthening the quality of services at drug shops is an alternative option. However, achieving quality services at drug shops is limited by multiple challenges including sub-optimal drug dosing, use of medicines not in national treatment guidelines [80], limited diagnostic capacity and inconsistent referral practices [55, 81], and the desire for profits [82]. Additionally, despite laws that provide for standards of operations, many drug shops operate illegally [83], with poor quality of health services [46, 84, 85], including inappropriate provision of parenteral artesunate [86]. This situation calls for policymakers to embark on programs of action to empower and strengthen regulatory frameworks and instruments in relation to drug shops [75].

Regardless of limitations, policy makers must acknowledge that drug shops are a popular point of care and can be mobilized to treat acute febrile illnesses among rural populations. In Tanzania [67] and Kenya [87, 88] interventions including provision of subsidized packs of pediatric ACT to retail outlets, training of retail outlet staff, and community awareness activities resulted in significant increases in ACT coverage for reported fevers. In-appropriate use of antimalarials by drug shops remains a concern. However, provision of rapid diagnostic tests for malaria in the private sector could minimize this problem as was demonstrated in a study in Ghana [89], where introduction of RDTs resulted in reduced dispensing of antimalarial to patients without malaria, and did not reduce prescribing of antimalarials to true malaria cases. Additionally, by applying principles of commercial marketing to social health problems, drug shops have the potential to serve as an effective mechanism for promoting socially beneficial behavior change [75]. Also banning drug shops because of poor quality of care concerns may run them underground, causing more harm than good. Key is finding the right balance, supporting drug shops to provide much needed services, but maintaining acceptable standards of care.

In conclusion, we report on delays in seeking appropriate care by caregivers of children eventually diagnosed with severe malaria. Lack of action for perceived mild fever contributed to delays in care. Social networks including grandmothers and mothers-in-law, whose influence often resulted in inappropriate treatment options, led to further delays. Despite the opportunity, drug shops have been shown to contribute to delay in seeking appropriate care [16], possibly explained by providing inappropriate care. Unfortunately, caregivers often sought care at public health facilities only when interventions at home or at drug shops had failed and illness had progressed from mild to severe. To address these gaps, there is need for a multipronged approach, starting with prioritization of health education programs sensitizing communities living in endemic areas, not only on recognition of symptoms of malaria, but emphasizing the significance of fever, irrespective of grade as an indicator of malaria [20]. For drug shops, there is need to define the role ofdrug shops in providing care to sick children in sub-Saharan Africa. Once defined, shop attendants should be trained and regulated to operate appropriately. For the public health sector, governments and partners need to direct efforts towards improving quality of hospitality services, a neglected area deserving attention.

## Supporting information

S1 Appendix In-depth interview guide. (PDF)

S2 Appendix Checklist for fulfillment of consolidated criteria for reporting qualitative studies (COREQ). (XLSX)

S3 Appendix Transcript for interview A325. (PDF)
