# Supplementary material for: Understanding the context of delays in seeking appropriate care for children with symptoms of severe malaria in Uganda
Source: PLoS One. 2019 Jun 5;14(6):e0217262. doi: 10.1371/journal.pone.0217262 (PMC6550380; doi:10.1371/journal.pone.0217262)
Supplement: S1 Appendix — (PDF) [file pone.0217262.s001.pdf]

Appendix 1: In-depth interview guide

**Patient initials:** |_____|_____|	**Respondent initials:** |_____|_____|	**Study ID:** |____|/|____|____|____|
**Date of interview:** |____|____| **day** |____|____| **month** |____|____| **year**		
**Start Time of interview:** |____|____| **hours** |____|____| **minutes** □ **am** □ **pm**		
**End Time of interview:** |____|____| **hours** |____|____| **minutes** □ **am** □ **pm**		
**Time taken to conduct interview:** |____|____| **hours** |____|____| **minutes** □ **am** □ **pm**		
**Name of interviewer:**		
**Location of interview:**		

**Respondent demographic information**	
**Age:** |_____|______| Years	**Gender:** □ Female □ Male
**Highest level of education:**	
**Occupation:**	

Introduction to in depth interview

"Hello my name is ____________________ and I am interested in interviewing you for this study. This study is about young children aged 6 months to 10 years. Children of this age often suffer from different kinds of diseases including malaria, diarrhea, pneumonia, and others. We would like to learn from you, which of these diseases mostly affect your children and how you recognize that the child is unwell, and how you treat or respond when you think the child is unwell. You have been chosen to participate in this study because your child fits in this age group and has been enrolled in this study. We would therefore like to learn from your experience. The interview will take about 50 minutes.

Do you have any questions? Do you agree to continue before we start?

Now I request that we all switch off our mobile phones or put them in silent mode so that we are not distracted.

Domain	Topics and Probes
**Information about the child under treatment**	a) How many children do you have aged between 6 months and 10 years?
	b) What birth order is this child?
	c) Does this child that has been admitted usually fall sick?
**Previous experience with treatment of malaria**	a) Is this the first time the child is suffering from malaria? If yes, proceed to next section.<br>*If the child has suffered from malaria in the past, talk about previous experience with treatment of malaria for this child.*<br><br>— *Probe for what malaria is, how they knew it was malaria, types of malaria including their causes and symptoms, how mild and complicated malaria is spread from one person to another, what people in their area talk about malaria and its cause, who first noticed that the child had malaria in the home, how they prevent malaria in the home.*<br><br>— *Probe for whether they think that malaria alone can cause complications in children including death, who they think is most at risk of malaria in the home and why, whether they thought that this child was at risk of getting malaria.*<br><br>— *If one of the symptoms mentioned is fever, probe for what fever is, types of fevers, what it means to them when a child has a fever, how they respond to each of these types of fevers and why, which fevers deserve attention and which ones do not.*<br><br>— *Probe for who they sought permission from before responding to the child's illness, who they consulted regarding the action to be taken and what advise were they given, whether this person was around when the child fell sick, what their relationship is with the people they consulted or sought permission from, whether they always seek permission from them or consult them before treating the child, what would happen if they did not seek permission or consult this person*<br><br>— *Probe for where they sought care, why they chose to see care there, who made the decision on where to seek care, what was done at the place where they sought care, whether they were satisfied with the services rendered and why and whether they would go back to that place to seek care.*<br><br>— *Probe for the costs they incurred while caring for the child during this illness, who catered for the costs, whether they were able to cater for all the necessary costs, whether they had control over resources for transport and other treatment related expenses or someone else had to decide.*<br><br>— *If caregiver used traditional herbs, probe for the name of the herb, why they chose to use it, where they got it from, whether it worked, how it worked, how they learnt about*

	the herb, whether its commonly used in their village.
**Experience with the episode of malaria that got the child admitted**	a) Now let us talk about the episode of malaria that got this child admitted. When did the child become unwell? Who in the home first noticed that the child was unwell? What did they see that made them realize that the child was unwell?<br><br>— *Probe for how the illness started, description of the symptoms that they saw, what they thought the child was suffering from when they saw these symptoms, whether they considered this normal or unusual, whether they thought the illness would result into severe disease and why.*<br><br>— *Probe for who they sought permission from before responding to the child's illness, who they consulted regarding the action to be taken and what advise were they given, whether this person was around when the child fell sick, what their relationship is with the people they consulted or sought permission from, what would happen if they did not seek permission or consult this person*<br><br>— *Probe for where they sought care, why they chose to see care there, who made the decision on where to seek care, what was done at the place where they sought care, whether they were satisfied with the services rendered, and why and whether they would go back to that place to seek care.*<br><br>— *If caregiver used traditional herbs, probe for the name of the herb, why they chose to use it, where they got it from, whether it worked, how it worked, how they learnt about the herb, whether its commonly used in their village.*<br><br>— *Probe for what other services are available near their homes where they can seek care for their child. Asks for whether they believe that the offered services near their homes could actually help their child or not and why.*<br><br>— *Probe for the costs they incurred while caring for the child during this illness, who catered for the costs, whether they were able to cater for all the necessary costs, whether they had control over resources for transport and other treatment related expenses or someone else had to decide.*<br><br>— *Probe for whether the health of the child is considered important compared to other needs in the home. If not, why? Probe for whether they have ever had to postpone providing care due to engagement in other activities. What activities? Who made the decision to prioritise these needs over attending to the child's health? Probe for the respondent has ever got in trouble for not following expectations of their husband/father/mother/community with regard to providing health care for the children. What happened?*
**Closing**	Is there anything else you think is important that you would like to add about treatment of malaria in children that we have not talked about?

CONTACT SUMMARY FORM	
**Interviewer to complete this form after the interview**	
**Study ID: |____|/|____|____|____|**	**Date : |____|____| day |____|____| month |____|____| year**
1. How would you describe the atmosphere and context of the interview (*Include interview location and how this may have affected responses)*?	
2. What were the main points made by the respondent during this interview?	
3. What new information did you gain through this interview compared to previous interviews?	
4. Was there anything surprising to you personally? Or that made you think differently?	
5. Were there any problems with the topic guide (e.g. wording, order of topics, missing topics) you experienced in this interview?	
