# Supplementary material for: Understanding the context of delays in seeking appropriate care for children with symptoms of severe malaria in Uganda
Source: PLoS One. 2019 Jun 5;14(6):e0217262. doi: 10.1371/journal.pone.0217262 (PMC6550380; doi:10.1371/journal.pone.0217262)
Supplement: S3 Appendix — (PDF) [file pone.0217262.s003.pdf]

INTERVIEWER: Let us move on to the illness that got him here.

A325: Hmm

INTERVIEWER: How did it start?

A325: It started like fever. He got very cold in the morning when I had gone to work. The mother of the children said that the child's body had become hot in the night and it seems the child is going to fall sick. You know you always get to know when the child is going to get sick.

INTERVIEWER: The child's body was hot.

A325: I said let me go to a place nearby and I will return later and we shall see what to do. Maybe we shall get him some drugs or something. I don't know. Sometimes he may get a bit sick and then recover. I went. When I was in the garden they came for me. They said hallo, the child you left with a hot body, at this very moment his body is very hot and they have covered him but the beddings they have covered him with seem like they have poured water on them.

INTERVIEWER: Eeh he was sweating a lot.

A325: But he was shivering. The body head had reduced and now they had started covering him so that he gets warm.

INTERVIEWER: What do they usually cover him with? Some people use mats.

A325: Bed sheets and blankets.

INTERVIEWER: So they had covered him and all the beddings were wet.

A325: He was feeling cold. I rushed to the clinic and got some drugs and gave him with a syrup. They told me that it was for the blood.

INTERVIEWER: Had you gone with the child to the clinic?

A325: I went with the child to the clinic. They said that the child needed blood. She suggested the syrup and I asked her how much it would cost. She told me that the syrup needed mixing. It was sealed and the drugs he was going to mix and what else…I have forgotten. He said that those things; the syrup I should give the child one spoon and also told me when to give the drugs. I went with the drugs. When I reached home I told my wife that they had told me that we should give the syrup this number of times and the drugs should be crushed and given to him. For these ones put in the cup and give the child to drink. Aah

INTERVIEWER: Then you gave the child. Did you get to know what drugs had been given?

A325: With those drugs, you know with things you did not study about, they usually fail you. Sometimes they give you and you think they will work. I left the responsibility of giving the child drugs to my wife. It was about 11am and we had started using those drugs and the temperature fell. It seemed like he was getting better. The syrup is even still at home. We stayed there and then the following morning, it was now two days using those drugs. I was working and they came for me and told me that the child seemed like he was getting better but they didn't know what had happened. Now we have our people like you

know (unclear words)….you will forgive me if you do not agree with them but for me because of the pain I used them. They told me that that medicine works for the children and explained how they gave their child. They said the child was ill.

INTERVIEWER: What kind of people were those?

A325: They were my neighbours. I told them that they had given me drugs for the illness but it was still remaining and he had not taken all of it. They told me that he had an illness that was inside. (Touches stomach to demonstrate). They are foreigners.

INTERVIEWER: Are they like traditional healers?

A325: Yes. So I went and when they checked the child they said there was nothing.

INTERVIEWER: How did they check? Were you present?

A325: Yes

INTERVIEWER: How did he check?

A325: He has things like a witch doctor. There is some grass that puts here or in the face. He asks for where the pain is. He touched here and told me to take the child where he can get blood because it seemed the blood was little. I will not lie to you that there are bones involved in this child's case. I thanked him because he would have asked me for money without helping me. I rushed and took him to the hospital.

INTERVIEWER: That very day.

A325: Yes.

INTERVIEWER: Did you take him to a drug shop?

A325: No. It was a government hospital. They told me that I had delayed. The child is dying because of lack of blood and water. I asked for what to do and they told me to go to Nsinzi HC IV. It is the people there that saved us by sending us to Iganga because at Nsinzi there was no blood. When I go to Nsinzi they told us that the child lacked blood. I asked them for what to do. They told me that they were sending me to Iganga. They said I should get a vehicle and leave very quickly. They said I should not take the child back home. I did not have money I should just go with the child. I boarded the vehicle. I told the people that had escorted me to go back and find something to sell and bring the money to me. I left.

INTERVIEWER: Was your wife one of the people that you told to go back home?

A325: No. It was my mother.

INTERVIEWER: It was your mother.

A325: Yes. My wife has a neonate that follows this one.

INTERVIEWER: Aah so she cannot move around.

A325: My sister in law is the one helping out here. I boarded the car from Nsinzi with the letter I had been given to take to Iganga. When I got inside the vehicle the other passengers told me eeh how long has the child been sick. I said only three days and this is

the fourth day but he has become a problem. They asked me where I was taking him and I told them Iganga. They asked why I was going to Iganga and I said to get blood. They told me not to bother. They suggested that I go to Mbale.

INTERVIEWER: Eeh so they were telling you to go to Mbale.

A325:They wanted me to go back yet I was half way the journey. I wondered how I would get to Mbale. I did not have money and the people I had told that I had gone to Iganga and that they should get money and bring it to Iganga will not find me there. I decided to just proceed to Iganga and if I did not find blood I would go to Nalufenya because I hear that they are good at treating. There was a man and woman seated in the front seat. They said that ooh it is true that if you want the child to recover you rush there. Do not even bother with Iganga. At Iganga you will suffer and go back home with the dead body of your child. I closely followed everyone that said anything concerning the child. We went up to the park. When I reached the park the child died.

INTERVIEWER: The child died!

A325: Yes. I saw that there was nothing left of the child. The people that were around asked where I was taking the child. I told them I was taking the child to hospital. They asked what the problem was and I said lack of blood. They asked where I was taking a dead child. They told me to take the child back home. They tried to stop me. I told them I did not have money to return home and that I was heading somewhere and I had sent some people to go back and get me some money. If the child is dying in my hands, I do not have money. Some kind people gathered 1000 shillings each and then there is a woman that gave me 5000 shillings and told me to buy a box or get in the car and take the child back home. She said it was clear that the child was no more. She said that I am a man and may not know about things concerning children. She said that she had had children and that I was bothering for nothing. Some people said that the child was still alive.
INTERVIEWER: That the heart is still beating.

A325: So I asked for their advice. I was going to Nalufenya. I was told that they always have blood. They told me that I may find that there is no blood and they asked how I was planning to move with the child. They collected some money for me and I got 10,000 shillings.

INTERVIEWER: That was in the park.

A325: Yes. So I was going back since the child was hopeless. The truth is that even when you touch his hand it was lifeless. There was a woman that was very kind and my prayer is that God gives her the things she needs the most. She just Intervieweratched the child from me. She came and asked what was happening. I told her the child was dying. She asked what the child was suffering from and then she grabbed the child and took a bodaboda and got lost. I told a bodaboda guy that the lady that had just left had gone with my child and I do not know where she is taking the child. I told him that I wanted to take the child back home because I had failed. He said that she had told them that I should take one of the bodabodas and find out where she had gone with the child. I got onto one of the bodaboda and told him to chase the woman because I did not know where she had taken the child. He said that she was taking the child to the clinic or to the hospital. I said I did not think there was a hospital that would manage the child. I said that I wanted to

take the child back home since things had failed. Then I realized we were slopping and approaching the woman. She asked the bodaboda man whether I was the father of the child and he said yes. I told her that I was the one and she told me to go in. She paid off the bodaboda riders and we got into the hospital. She rushed in with the child. The health workers that were there were also kind. They quickly measured the child. Lucky enough the blood that was available was for blood group C which is what the child needed.

INTERVIEWER: Eeh he was lucky.

A325: We went. The rest of the patients that were there had failed to find blood. I was lucky they also gave the child blood and I was given some drugs. Right now the child is recovering.

INTERVIEWER: Eeh

A325: It is because of that woman that I got here and when the child manages to stand on the ground I will be very happy.

INTERVIEWER: He will be fine.

A325: I would like her to come to my home and see the child. I did not think that he could get better but right now he is eating. He had not been eating and drinking. From the time he started falling sick everything you gave him he could not take. He started drinking yesterday and today. So I am very happy with this hospital. I will even tell the others that are badly off like I was to come straight here. If they do not know this place, I would be willing to bring them if they could pay for my transport. That is the happiness you see now. It is at this hospital that I have found the right thing and that is why I am happy. That is it ma'am.

INTERVIEWER: Eeh I'm glad you made it through.
